# Hygiene Railway Traveling

**Published:** 1865

**Authors:** 


Hygiene Railway Traveling.—Dr. Decaisure in his *Guide Medical et Hygienique du Voyageur* makes the following remarks on the precautions to be taken in traveling by rail in a hygienic point of view. The first danger to be avoided is, according to our author, catching cold by rushing out heedlessly to the railway carriage, as most people do, the moment the doors are opened, in order to get a good place. The traveler gets in panting for breath and exposed to draught on all sides, which is extensively hurtful to the constitution. He recommends the traveler to take some slight refreshment beforehand, the external air inhaled in the morning, in the open country being too keen for a fasting stomach. He considers as a most hurtful habit that of rushing out from the carriage to the refreshment room the moment the train stops, in order to gobble up as fast as possible, any quantity of food, no matter what, so as to be in time again for the start. According to our author it is infinitely more advisable to select something very light, such as a basin of broth or a sandwich, that can be taken quietly and without any hurry; otherwise the digestive functions might be seriously disturbed, especially by the mechanical action of the train in motion, or the stomach filled with ill-masticated food. You are aware that certain English physicians have drawn a lamentable picture of the various diseases which may be contracted during railway traveling. Dr. Decasisure does not subscribe to their opinion; on the contrary, he considers this mode of locomotion as exercising a favorable influence on the public health; the only real danger to be guarded against being colds from exposure to draught, and indigestions by hasty and immoderate meals.

